# Race and Alzheimer Disease Biomarkers: A Neglected Race

**DOI:** 10.1212/NXG.0000000000000574

**Published:** 2021-03-04

**Authors:** Alberto Lleó, Marc Suárez-Calvet

**Affiliations:** From the Memory Unit (A.L.), Department of Neurology, Hospital de la Santa Creu i Sant Pau, Biomedical Research Institute Sant Pau-Universitat Autònoma de Barcelona; Centro de Investigación Biomédica en Red Enfermedades Neurodegenerativas (CIBERNED) (A.L.), Madrid; Barcelonaβeta Brain Research Center (BBRC) (M.S.-C.), Pasqual Maragall Foundation, Barcelona; Servei de Neurologia (M.S.-C.), Hospital del Mar, Barcelona; IMIM (Hospital del Mar Medical Research Institute) (M.S.-C.), Barcelona; and Centro de Investigación Biomédica en Red de Fragilidad y Envejecimiento Saludable (CIBERFES) (M.S.-C.), Madrid, Spain.

Race is a social construct with profound consequences on health. Although race does not always segregate with genetic ancestry, some genetic variants are more frequently found in certain self-identified racial and ethnic groups. Differences in the epidemiology and the pathophysiology of Alzheimer disease (AD) and other neurodegenerative conditions have been described in certain racial groups. Unfortunately, most studies in the field of AD have mainly included non-Hispanic White (NHW) participants, with an underrepresentation of other populations. In the past few years, however, research on race and ethnic differences in AD has grown rapidly. Some studies indicate that African Americans (AA) have a greater risk for AD than NHW.^1^ The reasons hypothesized to account for this higher risk are certain genetic risk factors associated with AA, the higher prevalence of vascular risk factors in AA, and socioeconomic factors.^2^ In addition, some studies have reported a different pathophysiology and pattern of AD biomarkers. There is evidence that AA have lower concentrations of total-Tau, phosphorylated Tau, and neurofilament light in the CSF compared with NHW.^3^ The differences in AD pathophysiology in racial groups can be due to various factors, such as genetic variants associated with that race, but also with other environmental factors such as socioeconomic status, diet, or medical comorbidities. Another factor that could play a role in this association is a different innate immune response to AD pathology.

In this issue of *Neurology*® *Genetics*, Schindler et al.^4^ take advantage of the racial and ethnical diversity of the Knight ADRC cohort to describe differences in soluble TREM2 (sTREM2) concentrations in the CSF in AA and NHW. AA participants had lower CSF sTREM2 concentrations, a difference that was mainly driven by the higher frequency of certain *TREM2* coding variants associated with lower CSF sTREM2. These results remained significant after adjusting by age, sex, education, history of dementia, and *APOE*ε4 status. The authors also confirmed their findings in participants of the ADNI cohort.

TREM2 is an innate immune receptor of the immunoglobulin family that is expressed on the plasma membrane of microglia and other myeloid lineage cells. TREM2 is involved in microglia migration, proliferation, phagocytosis, and lipid sensing.^5^ Low frequency coding variants in *TREM2* increase the risk of AD.^5^ In mouse models of AD, impaired TREM2 function limits the microglia response around Amyloid-β (Aβ) plaques and leads to greater neuritic dystrophy.^5^ These observations are consistent with a model in which defective TREM2 function affects microglial response to Aβ plaques and increases neuronal and synaptic damage.

CSF sTREM2 reflects the amount of TREM2 competent signaling on the surface of microglia and can thus be used as a biomarker of the TREM2-mediated microglia response. CSF sTREM2 dynamically changes throughout the Alzheimer *continuum*, with higher levels in the later asymptomatic stages and early symptomatic stages of both sporadic and autosomal-dominant AD.^6,7^ It is important that lower CSF sTREM2 concentrations have been associated with faster cognitive decline in patients with AD,^8^ which supports the notion that sTREM2 can be used as a surrogate marker of microglial activity in AD. This observation also suggests that TREM2-mediated microglial response may have a beneficial effect in AD, at least at some disease stages.

The work of Schindler et al. has important clinical implications. First, if AA have lower CSF TREM2 concentrations, the question arises of whether AA may be less protected by TREM2-related microglial function. Second, if AA show different AD biomarkers, it is important to account for race when designing cutoffs in a diverse population. Third, because TREM2 is expressed in other tissue macrophages, *TREM2* coding variants associated with AA may also induce a different peripheral inflammatory response. The peripheral inflammatory response is known to influence cognitive disorders and progression of AD,^9^ which could also have an impact on AD pathophysiology.

Finally, the findings reported by Schindler et al. may have therapeutic implications. Therapies that target TREM2 have already reached clinical phase 2 (NCT04592874). There is an ongoing clinical trial with a TREM2 activating antibody that enhances the protective functions of microglia in a mouse model of AD.^10^ It will be important to investigate whether TREM2 therapies have a different effect in participants with lower TREM2 function, irrespective of self-identified race. If true, then it would be key to monitor TREM2 function and adjust the dose accordingly. This would require the development of accurate assays to monitor microglial function through the course of the disease.

In summary, the study by Schindler et al., adds on an important and under-investigated aspect of AD. Because clinical trials grow and enroll patients with different races and ethnicities, it will be important to take both into account when interpreting outcomes.
